# Adjusting Historical Costs for Inflation with the Use of Standardized Automated Tools

**DOI:** 10.2147/CEOR.S476426

**Published:** 2024-09-14

**Authors:** Jason Robert Guertin, Naomi Hope Chouinard, Amélie Forget, Lucie Blais

**Affiliations:** 1 Centre de recherche du CHU de Québec-Université Laval, Quebec City, Canada; 2 Department of Social and Preventive Medicine, Faculty of Medicine, Université Laval, Quebec City, Canada; 3 Centre de recherche en organogénèse expérimentale de l’Université Laval/LOEX, Quebec City, Canada; 4 Faculty of Pharmacy, Université de Montréal, Montréal, Canada; 5 Centre de recherche du Centre intégré universitaire de santé et de services sociaux du Nord-de-l’Île-de-Montréal, Montréal, Canada

## Introduction

Interest in cost analyses and broader economic evaluation has been steadily rising in recent decades. Unfortunately, training and capacity in these fields has failed to follow the demand which has led to an increase in researchers, with sometimes minimal know-how, conducting these studies. Of course, experts in the field have provided multiple textbooks^1^ as well as guidelines^2^,^3^ to help novice researchers align their work to the high standards of the field but, too often, methodological errors remain present.

Cost analyses are one such type of studies that are prone to errors.^4^,^5^ Indeed, cost analyses are generally viewed to be relatively simple, at least in comparison to other types of studies conducted within the Health Economics field. However, they contain their own particularities which may be omitted by less experienced researchers. This risk is particularly true given the rise in interest for real-world data and real-world evidence based on medico-administrative data. To some extent an observational cost study based on longitudinal data is conceptually very similar to an observational effectiveness study based on longitudinal data. In both cases, researchers must aggregate outcomes (whether they be an effectiveness measure or a cost) over the examined period within the different exposure groups. That being said, additional steps, unique to cost studies, must be considered, namely the adjustment for inflation that is required when pooling data over long periods of time. The omission of adjusting costs for inflation can lead to erroneous interpretation of data, especially when working with older cost inputs. For example, 100 CAD in 2006 is equivalent to 147.40 CAD in 2024 when adjusted for inflation, ie, a 47.4% increase.^6^

Of course, we recognize that available web-based tools allow the adjustment for inflation and even account for differing purchasing power parities between countries.^7^ However, the value of these tools may be limited when conducting analyses on medico-administrative databases as access to the internet may be restricted within data access hubs and/or impractical if the adjustment must be repeated numerous times. Considering those issues, our team set forth with the aim of providing a novel option that could easily adjust numerous cost inputs at the same time. To do so, we created a validated automated SAS script (ie, a SAS macro; SAS Institute, Cary, NC) that could be downloaded and used by researchers to adjust large quantities of cost inputs for inflation over time.

## Methods

The creation and validation of our SAS macro followed a 3-step process: 1) Creation of two independent automated SAS scripts; 2) Validation on synthetic and empirical databases; and 3) Retroaction from potential users.

### Creation of the Automated Script

Our team established two criteria that the SAS macro needed to comply with: 1) having users specify annual consumer price indices (or other indices, if relevant) that would be used to adjust the cost inputs in the datasets; and 2) once indices and specific parameters would be made available to the macro, the macro would create a new variable which contains the inflation-adjusted values.

Aligned with CADTH’s Guidance document for the costing of health care resources in the Canadian setting,^2^ we used all-item consumer price indices available from Statistics Canada.^6^

Once we established these two principles, two analysts (NHC and AF) from two different laboratories each independently created a macro; the macros’ ability to adequately adjust cost data for inflation was examined by our team within the validation process.

### Validation on Synthetic and Empirical Data

Validation of the two macros was conducted on three distinct datasets, one common synthetic dataset created by our team and two empirical datasets obtained from two data repositories available to researchers in the province of Quebec (Canada), ie, the Institut de la statistique du Québec and reMed.^8^ The first database contains administrative data compiled by the Québec government. reMed is a research database containing information on medication prescribed to individuals and dispensed by community pharmacies for a sample of Québec residents.

The synthetic dataset was comprised of three types of variables and designed to mimic real-world data: 1) unique identifier; 2) date (ie, time of billing); and 3) costs related to the billing. All entries were generated randomly. Once the macros are applied to the raw data, it produces a fourth variable providing the inflated cost data. In order to test the two macros, analysts examined the concordance between the results obtained by their macro and from those obtained by manually coding the adjustment for inflation (defined as the gold standard in this comparison); values were compared using the SAS PROC COMPARE procedure. Coding errors in the macros, if any, were corrected at this stage.

Validation of the two revised macros was then conducted on sample datasets from previously identified empirical sources. These sample datasets (one per source) contained at least 1000 different individuals and at least 100,000 distinct observations; concordance of results obtained by both macros within each sample dataset was once again examined using the PROC COMPARE SAS procedure.

This project was authorized by the CHU de Québec-Université Laval’s Research Ethics Board (authorization number #MP-20-2023-6693) and complied with appropriate data protection and privacy regulations.

### Retroaction from Potential Users

Once both macros were validated, potential users, identified from the Quebec Drug Research Network’s list of members, who were not involved with their creation were asked to provide their opinion on the code and their perception in regards ease of using the macros. Users’ comments were used to finalize the SAS macros and instructions.

## Results

**Figure 1:**
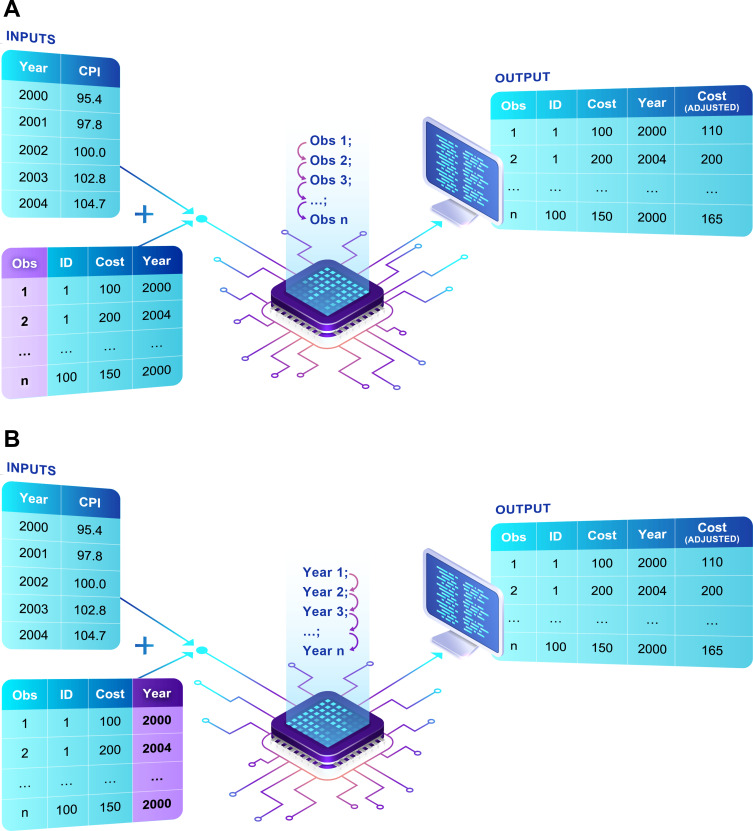
(**A**) Graphical representation of macro 1. (**B**) Graphical representation of macro 2.

**Notes**: Macro 1 combines two datasets, one containing the raw costs and the second containing the yearly consumer price index (CPI) values. The output of the macro is a dataset containing both the raw costs and the inflated costs. Macro 2 uses macro variables specific to the target consumer price index (CPI) values and applies these macro variables to the raw dataset to create a new variable containing the inflated costs.

Two distinct fully functional macros were created by two analysts. Despite common instructions, assessment of the codes showed that their design differed substantially. Main differences concerned how adjustment for inflation was conducted (ie, either sequentially through the different study observations [macro 1] or within a yearly loop system [macro 2]) and how the consumer price indexes is provided to the macro (ie, either by the user referring to a distinct dataset [macro 1] or by the user creating new macro-related variables [macro 2]) (Figure 1). Testing the macros on the synthetic and empirical datasets did not show any error with the code and resulted in identical inflated costs. In addition, testing highlighted that both versions could be of value for potential users depending on the volume of cost inputs to be adjusted and on users’ experience with SAS.

Following retroaction from potential users we made slight modifications to the code, ie additional instructions within the script and the inclusion of automated error warnings. Final versions of the macros and explanations on how to select between the two are made available in the Supplementary File 1.

## Discussion

We developed two distinct SAS macros that automate the adjustment for inflation of cost data within medico-administrative databases. Interestingly, we initially planned to create and validate two distinct macros to conduct the same task with the goal to identify the optimal macro. However, through our validation process, we realized that both macros could be of value to users depending on their level of experience with cost studies and with SAS programming. As such, we decided to provide both macros within this manuscript allowing users the opportunity to select their preferred version. In fact, as macro 2 creates macro-related variables, it may be particularly useful for users needing to adjust cost variables over a few years. It could also be convenient for novice SAS users as it is unnecessary to create an additional dataset for CPI values. For adjustment spanning over a significant period, or for repeated adjustments, macro 1 may be optimal for users as it requires a distinct dataset containing CPI values created by the user. Experienced users may also prefer macro 1 as the same CPI file can be used in various cost projects.

In conclusion, we provide two validated and freely available macros designed to adjust cost data for inflation. Seeing as demand for expertise within Health Economics is expected to continue to increase beyond current capacities, knowledge translation endeavours accounting for the investigators’ level of expertise, such as these, are needed to help improve the overall quality of future cost studies. Future work should focus on expanding the availability of automated tools such as this macro to other programming software for broader use.
